# How does mindfulness relate to benign/malicious envy? The mediating role of resilience, internal locus of control and self-esteem

**DOI:** 10.3389/fpsyt.2022.878690

**Published:** 2022-08-30

**Authors:** Xia Dong, Xiaojun Li, Xinsheng Jiang, Yanhui Xiang

**Affiliations:** ^1^School of Educational Science, Hunan Normal University, Changsha, China; ^2^School of Teacher Education, Nanjing Xiaozhuang University, Nanjing, China; ^3^Cognition and Human Behavior Key Laboratory of Hunan Province, Hunan Normal University, Changsha, China

**Keywords:** mindfulness, internal locus of control, resilience, self-esteem, benign/malicious envy

## Abstract

The present study aimed to explore the roles of resilience, internal locus of control, and self-esteem in the link between mindfulness and benign/malicious envy (BE/ME). Nine hundred ninety-one participants (299 males, 692 females; mean age = 19.05 ± 1.54) completed a survey that assessed mindfulness, internal locus of control, resilience, self-esteem, and BE/ME. The results suggest that resilience, internal locus of control, and self-esteem independently mediate the relationship between mindfulness and BE/ME. Additionally, “internal locus of control → resilience” and “self-esteem → resilience” play chain mediating roles in the relationship between mindfulness and BE/ME. Namely, mindfulness is positively associated with resilience *via* improving internal control and self-esteem, thereby inhibiting malicious envy and promoting benign envy. The present study advances our knowledge of the mindfulness reperceiving theory, and thus provides a new explanation for the inhibition of negative emotions from the perspective of resilience, internal locus of control, and self-esteem.

## Introduction

Mindfulness is generally defined as an individual's purposeful and uncritical focus on their current internal and external experiences, including emotions, cognition, physical feelings, and sensory stimuli (1). The tendency to experience an internal state of mindfulness is called trait mindfulness (2), and it is often measured using self-reported methods such as the Mindful Attention Awareness Scale (MAAS) (3). Several studies have also used the MAAS to explore the relationship between mindfulness and negative emotions (4–6). Mindfulness is linked with a lessening of envy (7). Envy consists of the negative emotions that occur when people realize that they lack the advantages, achievements, and properties of others (8). It has two forms that differ in their motivational nature: benign/malicious envy (BE/ME) (9). Of these, BE elicits self-elevating motivation and can be a positive phenomenon (10, 11), while ME elicits a motivation to destroy the advantages of others (10, 12, 13). Envy, particularly ME, may trigger social violence and criminal behavior (14, 15), and it is, therefore, important to further discuss mechanisms that may effectively inhibit the negative effects of envy. Prior research has provided preliminary evidence that mindfulness is positively associated with BE, but negatively with ME (6). Therefore, based on the mindfulness reperceiving theory, this article intends to replicate and expand the results of the prior literature by confirming the association between mindfulness and BE/ME and investigating the mediating roles of resilience, internal locus of control, and self-esteem.

Resilience is an ability or trait by which people effectively adapt to adversity (16, 17). As a trait, it explains why some people are more courageous in the face of adversity, while others are overwhelmed by it and even develop serious psychological problems (18). Prior studies have indicated that the mediating link between mindfulness and both BE/ME is resilience (7). This means that resilience acts as a positive psychological resource for effectively coping with envy. Importantly, resilience is composed of protective factors originating from the individual, their family, and society (19). Internal locus of control and self-esteem are typical protective factors of resilience that arise from the individual (20). The present study further explored the mediating role of resilience, internal locus of control, and self-esteem in the link between mindfulness and BE/ME.

Internal locus of control is defined as a person's belief that they control their own life (21). People with internal locus of control feel that their life outcomes are determined by their own actions and personal characteristics (22). Mindfulness is positively related to internal locus of control. Some theorists have proposed the mindfulness reperceiving theory: reperceiving has been found to help people choose behaviors that are consistent with their needs, interests, and values and to make them more likely to believe in their abilities (23). Internal locus of control helps people have confidence in their behavior, abilities, and attributes and make choices that meet their needs (22). Therefore, mindfulness is linked with a higher internal locus of control, and this relationship has been borne out by empirical research (20, 24).

Some studies have also proposed that the internal locus of control should be understood to consist of perceived control (25). Perceived control is the belief that an individual determines their own internal state and behavior (26). Perceived control is also one of the conditions for envy differentiation (11, 27). The higher an individual's perceived level of control, the easier it feels for them to change their disadvantageous situation, and the more likely they are to experience BE rather than ME (28). Therefore, internal locus of control may be associated with a tendency toward BE/ME. Specifically, internal locus of control may be a mediating mechanism between mindfulness and BE/ME. Additionally, internal locus of control is a protective factor of resilience (29, 30). This implies that mindfulness may also be positively associated with resilience by improving the internal locus of control, thereby indirectly impacting envy in either its benign or malicious form.

Furthermore, self-esteem is defined as an individual's general sense of their own value (31). According to the mindfulness reperceiving theory, reperceiving is a change in perspective that encourages people to keep an open and objective attitude toward their current experience (23). This means that people with higher mindfulness are less concentrated on negative feelings and thoughts; reperceiving is thus associated with high self-esteem (32). A majority of studies have also suggested that mindfulness is positively linked to self-esteem (33–35).

Additionally, research has demonstrated a link between self-esteem and BE/ME (36). People with low self-esteem have cognitive biases and often have a negative view of self (37). Thus, to avoid losing their valuable self-esteem resources after a negative upward social comparison, they may be particularly prone to use hostile strategies, and therefore more inclined to experience ME (36). Therefore, we inferred that self-esteem is negatively linked with BE/ME. Self-esteem, too, is a protective factor of resilience (29). Mindfulness may, therefore, also positively link resilience to BE/ME by promoting self-esteem.

Based on the above literature and the mindfulness reperceiving theory, we proposed four hypotheses: (1) Mindfulness is indirectly related to BE/ME through the mediating role of internal locus of control. (2) Mindfulness is related to BE/ME through the chain mediating mechanism of “internal locus of control → resilience”. (3) Mindfulness is indirectly related to BE/ME through the mediating role of self-esteem. (4) Mindfulness is related to BE/ME through the chain mediating mechanism of “self-esteem → resilience”.

## Methods

### Participants and procedures

Nine hundred ninety-one participants (299 males and 692 females) were selected from the eastern and coastal regions of china by random sampling and cluster sampling. participants' ages ranged from 17 to 26 (*m* =19.05, *sd* = ±1.54). They completed hardcopy informed consent forms and received compensation of 30 yuan after completing all questionnaires. This study was approved by the ethics committee of the author's university. Two exclusion criteria were used. First, a survey was excluded if more than 2/3 of the questions were not filled out. Second, a questionnaire was excluded if all the questions had the same answer, as this indicated that the participant did not answer them carefully. based on these two exclusion criteria, 64 questionnaires were excluded. Moreover, for *power* = 0.95, the required sample size for this study is 400. The sample size of this study was 991 (i.e., >400) meeting the requirement. It should noted that the data for the current study were from an ongoing project named “Philosophy and Social Science Project of Hunan Province of China”, some of the data have been used in previous studies (6, 7, 11, 13, 38).

The questionnaires included a short demographic survey and the MAAS, the Locus of Control Scale (LCS), the Connor-Davidson Resilience Scale (CD-RISC), the Rosenberg Self-Esteem Scale (RSES), and the Benign and Malicious Envy Scale (BEMAS). The participants completed all the questionnaires in about 40 min. Numerous studies have proven the effectiveness of this procedure (38–40).

### Measures

Brown and Ryan (1) devised the MAAS to measure mindfulness. It consists of 15 items (e.g., “I forget a person's name almost as soon as I've been told it for the first time.”). All items are answered on 6-point scale (1 = *almost always*, 6 = *almost never*), with higher scores meaning higher levels of mindfulness. Studies have shown this scale to be highly reliable with Chinese participants (41) (Cronbach's α = 0.86). Cronbach's α = 0.79 for this scale in the current study.

The BEMAS was devised by Lange and Crusius (10). It consists of 10 items, with 5 items for each of the 2 subscales (BE/ME). A representative item from the BE subscale is, “Envying others motivates me to accomplish my goals.” Meanwhile, the ME subscale included items such as, “I feel ill will toward people I envy.” Participants indicated their agreement on a 6-point Likert-type scale (1 = *almost disagree*, 6 = *almost agree*). Higher scores indicate higher levels of BE/ME. Many studies have demonstrated the validity of this scale for Chinese participants (6, 13, 39). Cronbach's α = 0.81 for the BE scale and 0.85 for the ME scale in the current study.

The CD-RISC was developed and revised by Campbell-Sills and Stein (17). It is composed of 10 items (e.g., “Tries to see the humorous side of problems”) and is a 6-point scale (1 = *almost disagree*, 6 = *almost agree*). Higher scores indicate stronger resilience. Xiang et al. (38) have confirmed its reliability with Chinese participants. Cronbach's α = 0.89 for this scale in the current study.

The LCS was compiled by Levenson (21) and divided into three dimensions, namely Internal Locus of Control, Powerful Others, and Chance. It has 24 items, 8 items of which measure internal locus of control. A representative item is, “Whether I can be a leader mainly depends on my ability.” Participants rated each item on a 6-point scale (1 = *almost disagree*, 6 = *almost agree*). Higher scores indicate a higher internal locus of control. Studies have confirmed its reliability with Chinese participants (42) (Cronbach's α = 0.71). The Cronbach's α of this dimension was 0.77 in the current study.

The RSES was compiled by Rosenberg (31) and includes 10 items. A sample item is “I take a positive attitude toward myself.” The scale uses a 4-point scale (1 = *strongly disagree*, 4 = *strongly agree*). Higher scores indicate higher levels of self-esteem. Kong and You (43) have confirmed its reliability with Chinese participants. Cronbach's α = 0.89 in this study.

### Data analysis

First, the possibility of common method bias (CMB) was analyzed. Second, a measurement model was established to determine whether the observed variables represent the latent variables well. Prior studies have shown that when using a structural equation model to construct a measurement model, the items can be packaged to divide the factor loadings (44). Specifically, the inter-item balance method was used to divide mindfulness and resilience into three parcels, and to separate internal locus of control, self-esteem, and BE/ME into two parcels. The effectiveness of this method has been verified in previous studies (39, 40, 43). Third, according to the good fit of the measurement model, the structure model was established, and some indexes (e.g., the Chi-Square Statistics) were adopted as indicators to evaluate goodness of fit (42–44). The bootstrapping method was then used to test the mediating role of resilience, internal locus of control, and self-esteem. Finally, a cross-gender stability analysis was used to examine gender differences.

## Results

### Common method biases

Given that this study was based on a questionnaire survey method, Harman's single factor test was used to measure CMB (45). The common factor was set to 1 for confirmatory factor analysis. The results indicated that the fitting index was not ideal ($\chi_{/\text{df}}^{2}$ = 8.015, RMSEA = 0.084, NFI = 0.451, GFI = 0.608, CFI = 0.483, SRMR = 0.089), demonstrating that there was no serious CMB in the data.

### Measurement model

Latent variables in the measurement model included mindfulness, resilience, internal locus of control, self-esteem, and BE/ME; there were 14 observed variables. The results showed that the data were suitable for the established models [χ^2^_(62,991)_ = 157.950, *p* < 0.001; RMSEA = 0.040; SRMR = 0.028; CFI = 0.985; AIC = 243.950; ECVI = 0.246]. Furthermore, all variables were significantly related; Table 1 includes the *M* (mean), *SD* (standard deviation), and correlations of mindfulness, resilience, internal locus of control, self-esteem, and BE/ME.

**Table 1:** Descriptive statistics and correlations for major measures.

	***M***	***SD***	**1**	**2**	**3**	**4**	**5**
1 MF	60.91	9.08	1.000				
2 RI	34.74	5.53	0.326^***^	1.000			
3 SE	29.38	4.63	0.300^***^	0.498^***^	1.000		
4 ILC	31.76	3.85	0.204^***^	0.400^***^	0.368^***^	1.000	
5 BE	23.35	3.59	0.122^***^	0.369^***^	0.292^***^	0.388^***^	1.000
6 ME	11.01	4.49	−0.281^***^	−0.308^***^	−0.274^***^	−0.266^***^	−0.205^***^

MF, Mindfulness; RI, Resilience; SE, Self-Esteem; ILC, Internal Locus of Control; BE, Benign Envy; ME, Malicious Envy;

*** *p* < 0.001.

### Structure model

**Figure 1:**
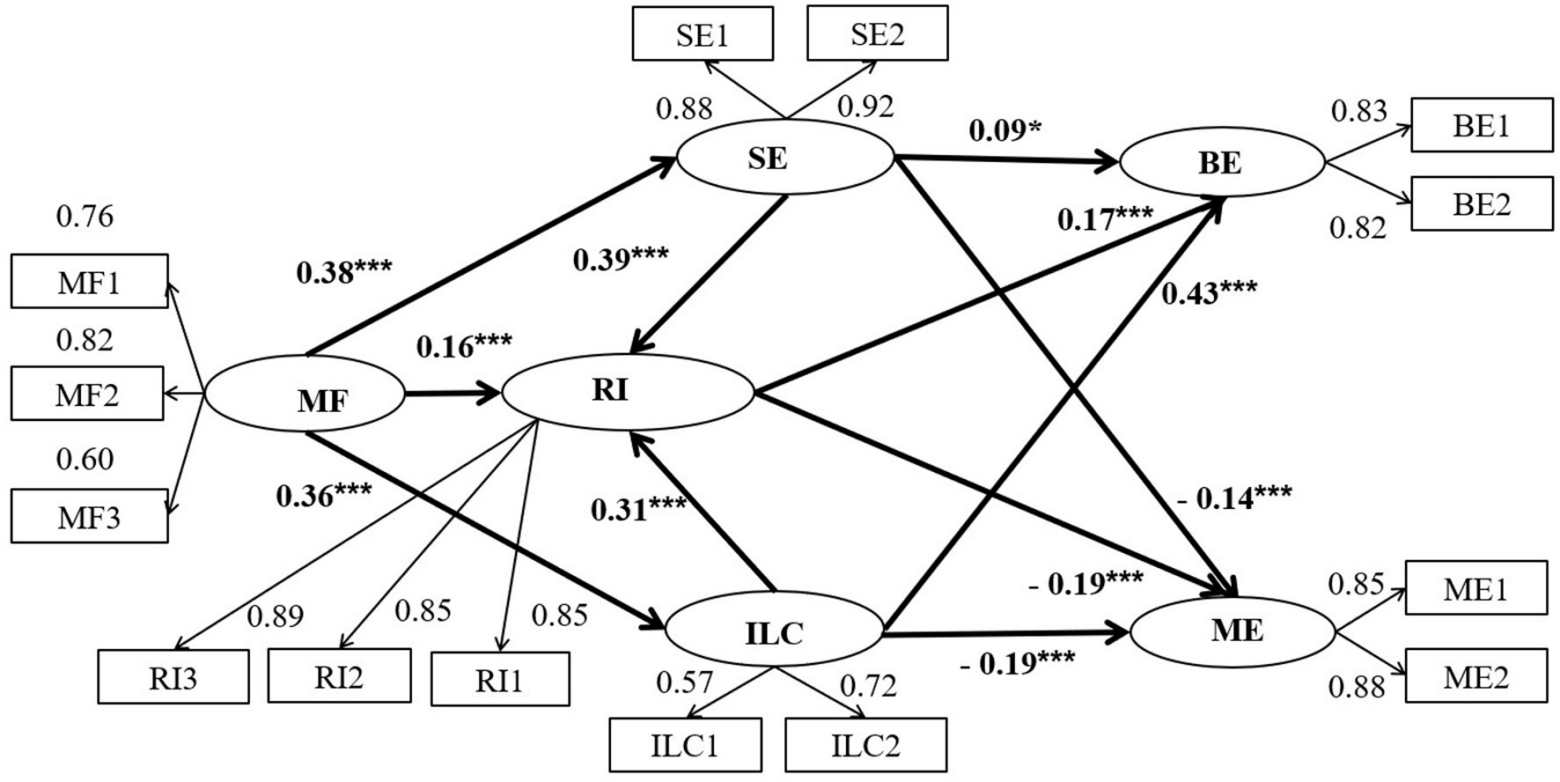
The mediation model factor loadings are standardized. MF1, MF2 and MF3 represent 3 parcels of Mindfulness, ILC1 and ILC2 represented 2 parcels of Internal Locus of Control, SE1 and SE2 represent 2 parcels of Self-Esteem, RI1, RI2 and RI3 represent 3 parcels of Resilience, BE1 and BE2 represent the two parcels of Benign Envy, ME1 and ME2 represent the two parcels of Malicious Envy. *p < 0.05, **p < 0.01, ***p < 0.001.

Once it was shown that the measurement model fit well, Structure **Model 1** was established to consistent with the hypotheses (see Figure 1). **Model 1** results indicated that each indicator matched well (see Table 2) [**Model 1**: $\chi_{\left(66,991\right)}^{2}$ = 340.310; *p* < 0.001; RMSEA = 0.065; SRMR= 0.069; CFI = 0.958; AIC = 418.310; ECVI = 0.423]. Therefore, **Model 1** was chosen as the final structural model.

**Table 2:** Fit indices of structure **Model 1**.

	**χ^2^**	***df***	**RMSEA**	**SRMR**	**CFI**	**AIC**	**ECVI**
Model 1	340.310	66	0.065	0.069	0.958	418.310	0.423

RMSEA, Root-Mean-Square Error of Approximation; SRMR, Standardized Root-Mean-Square Residual; CFI, Comparative Fit Index; AIC, Akaike Information Criterion; ECVI, Expected Cross-Validation Index; the same below.

### Testing of mediating variables

Bootstrap estimation was used to test the validity of the mediating variables (i.e., resilience, internal locus of control, and self-esteem). 1,000 bootstrap samples (*N* = 991) were selected from the raw data by random sampling. The results indicated that all the mediating variables played a significant role, with 95% CIs (see Table 3). Among them [95% CIs (0.004, 0.037)/95% CIs (−0.040 to −0.006)], internal locus of control [95% CIs (0.070, 0.159)/95% CIs (−0.096 to −0.011)], and self-esteem [95% CIs (0.001, 0.049)/95% CIs (−0.062 to −0.007)] played significant mediating roles in the link between mindfulness and BE/ME. Importantly, mindfulness had an indirect and significant effect on BE/ME through the chain mediating paths of “internal locus of control → resilience” [95% CIs (0.005, 0.025)/95% CIs (−0.024 to −0.006)] and “self-esteem → resilience” [95% CIs (0.005, 0.031)/95% CIs (−0.028 to −0.006)].

**Table 3:** Standardized indirect effects and 95% CIs.

**Model pathways**	**Estimated**	**Lower**	**Upper**
MF → RI → BE	0.018^**^	0.004	0.037
MF → RI → ME	−0.019^**^	−0.040	−0.006
MF → ILC → BE	0.103^***^	0.070	0.159
MF → ILC → ME	−0.042^**^	−0.096	−0.011
MF → SE → BE	0.022^*^	0.001	0.049
MF → SE → ME	−0.032^**^	−0.062	−0.007
MF → ILC → RI → BE	0.013^**^	0.005	0.025
MF → ILC → RI → ME	−0.013^**^	−0.024	−0.006
MF → SE → RI → BE	0.016^**^	0.005	0.031
MF → SE → RI → ME	−0.017^**^	−0.028	−0.006

MF, Mindfulness; RI, Resilience; BE, Benign Envy; ME, Malicious Envy; ILC, Internal Locus of Control; SE, Self-Esteem.

* *p* < 0.05,

** *p* < 0.01,

*** *p* < 0.001.

### Gender differences

The independent sample *T-*test was used to determine whether there were differences in gender between the six latent variables. The results showed that there were no significant gender differences in mindfulness [*t*_(991)_ = 0.192, *p* = 0.848], self-esteem [*t*_(991)_ = 1.162, *p* = 0.246], internal locus of control [*t*_(991)_ = 0.907, *p* = 0.365], or BE [*t*_(991)_ = −1.77, *p* = 0.077]/ME [*t*_(991)_ = −0.671, *p* = 0.502]. The gender difference in resilience was significant [*t*_(991)_ = 2.999, *p* = 0.003], with men scoring higher than women.

To test the gender stability of the structural model, a cross-gender stability analysis was performed. According to the basic constraint that factor loadings, error variances, and structure variances should be kept unchanged, two models were established, one of which allowed for an unconstrained structure path, while the other restricted the structure coefficients of the two genders to be equal (46). The model results indicated that there was a significant difference between the two [Δ $\chi_{\left(31,991\right)}^{2}$ = 68.728, *p* < 0.001]. Meanwhile, all fitting indicators of the model reached the standard of fitness (see Table 4). Furthermore, the CRD (absolute value range: >1.96) was used as an indicator to further explore the structural cross-gender stability (47).

**Table 4:** Comparison of unconstrained and constrained structural path models.

	**χ^2^**	**df**	**CFI**	**RMSEA**	**SRMR**	**AIC**	**ECVI**
Unconstrained SP	434.950	140	0.955	0.046	0.074	574.950	0.581
Constraint SP	503.678	171	0.949	0.044	0.075	581.618	0.588

The results showed that there was a significant gender difference in the structure paths of mindfulness → resilience (CRD _MF → *RI*_ = −2.233; *β*_men_ = 0.291, *p* < 0.001; *β*_women_ = 0.101, *p* = 0.031) and internal locus of control → resilience (CRD _ILC → *RI*_ = −2.174; *β*_men_ = 0.189, *p* = 0.013; *β*_women_ = 0.373, *p* < 0.001). Meanwhile, there were no significant differences in the structural paths of all other variables (CRD _MF → *SE*_ = 0.423, CRD _MF → *ILC*_ = −0.738, CRD _RI → *BE*_ = −0.306, CRD _SE → *BE*_ = 1.249, CRD _ILC → *BE*_ = −0.608, CRD _SE → *RI*_ = 1.326, CRD _RI → *ME*_ = 0.001, CRD _SE → *ME*_ = 0.778, CRD _ILC → *ME*_ = −0.259).

## Discussion

This article aimed to reveal the mechanisms by which resilience, internal locus of control, and self-esteem mediate the association between mindfulness and BE/ME. The results suggest that mindfulness effectively promotes the occurrence of BE and inhibits the occurrence of ME through resilience, internal locus of control, and self-esteem. Thus, this study further expands the theoretical basis of the mindfulness reperceiving theory. Interestingly, the comparison of cross-gender models found that there are gender differences in the path of “mindfulness → resilience” and “internal locus of control → resilience”.

First, unlike previous studies, our results reveal that mindfulness is positively correlated with BE. A possible reason for this relationship is that BE has a positive side. Prior studies have found that BE motivates people to narrow the gap between themselves and the envied (48, 49). In addition, the mindfulness reperceiving theory posits that mindfulness helps people focus more on their current experience and can break rigid responses (23), thus improving cognitive flexibility (50, 51). Therefore, mindfulness gives people strong cognitive flexibility, leading to less resentment and less sense of inferiority, making the individual more prone to experience BE. However, although our research shows that there is a positive side to BE, it must be acknowledged that BE is still a negative emotion. BE is also correlated with undesirable psychological outcomes in terms of personality. For example, Lange et al. (12) have found that BE is linked with the Dark Triad of personality. Therefore, future research needs to further explore the relationship between mindfulness and BE.

Second, the mediation analysis results reveal that mindfulness promotes BE and inhibits ME by strengthening the internal locus of control, validating **hypothesis 1**. One possible explanation is that mindfulness helps people make choices that meet their own needs (23) and makes them more confident in their own abilities (20, 24), thereby causing them to experience a greater sense of internal locus of control. Therefore, such people are more likely to experience BE than ME. Moreover, this finding indirectly suggests that internal locus of control is indeed equivalent to perceived control to some extent (25), thus influencing BE/ME.

In addition, the results also indicate that mindfulness relates to BE/ME through the chain mediating mechanism of “internal locus of control → resilience,” supporting **hypothesis 2**. This is probably because internal locus of control is linked to positive adaptation to adversity (20). Mindfulness motivates people to be more confident in their abilities (23), and thus enables them to actively adjust to adversity (52). As a result, they are more likely to experience BE than ME.

The mediation analysis results reveal that mindfulness indirectly predicts BE/ME through self-esteem, validating **hypothesis 3** and the mindfulness reperceiving theory. This is consistent with the fact that mindfulness increases self-esteem, thereby inhibiting negative emotions (34). Mindfulness is linked with greater cognitive flexibility and a lessened focus on negative thoughts (23), leading to higher self-esteem (53). Additionally, our research supports a positive link between self-esteem and BE. This finding is also consistent with that of Li and Xiang (13). Therefore, people with low self-esteem are more likely to experience ME rather than BE.

Importantly, mindfulness relates to BE/ME through the chain mediating mechanism of “self-esteem → resilience,” supporting **hypothesis 4**. That is to say, mindfulness improves an individual's self-esteem, thereby enhancing their resilience, and thereby promoting BE and reducing ME. One reason for this is that mindfulness helps people adjust their perceptions of negative experiences through reperceiving (23), thereby increasing self-esteem. Meanwhile, mindfulness is associated with positive self-assessment (54), and therefore with higher self-esteem (35, 55), and thus mindful individuals tend to positively adjust and adapt to adversity (33, 34), promoting their resilience (35, 55–57). Therefore, such individuals are more likely to experience BE than ME.

Interestingly, the cross-gender model analysis results showed that men have higher mindfulness, and thus more resilience, than women. This is consistent with prior studies, which have shown that men are more resilient than women in the face of adversity (58, 59). Men with higher levels of mindfulness are better able to actively adapt to adversity, and therefore are more resilient than women. However, women have higher internal locus of control, and thus more resilience. This supports previous studies that showed that women have a higher internal locus of control than men (60), and thus have higher resilience.

This article has some limitations. First, the participants were all Chinese youths. Future research should examine the mechanisms of mindfulness and BE/ME in groups of different ages and cultural backgrounds. Second, the structural equation model can only infer possible causal relationships between variables. Future research should adopt longitudinal research and experimental methods to explore more deeply the causality linking mindfulness and BE/ME. Third, the gender ratio of this study was unbalanced, and future studies should use samples that are more gender-balanced.

Despite the above limitations, this study still has some practical implications. First, this study shows that the further development of malicious envy can be avoided by using mindfulness to enhance resilience, internal control, and self-esteem. This finding might be useful for educators and employers to improve group performance in classrooms or workplaces. For example, educators can teach their students a mindful approach that increases their positive resources (i.e., resilience, internal locus of control, and self-esteem), so that they can avoid the painful experience of envy, especially malicious envy, and feel more satisfied with their lives (6). Meanwhile, in order to avoid malicious envy among employees, employers can also carry out proper training activities and use mindfulness to improve employees' resilience, internal locus of control, and self-esteem, thereby stimulating better work performance. Second, this study also found that mindfulness improves resilience through positive associations between internal locus of control and self-esteem, and helps to prevent individuals from developing malicious envy. This indicates that resilience, internal locus of control, and self-esteem are not completely independent, and they forestall the development of malicious envy through positive interactions with each other. Thus, educators and employers should focus on the development of the individual's overall positive resources (i.e., resilience, internal locus of control, and self-esteem) in the process of education or training, which can improve resilience and thereby avoid the negative effects of malicious envy.

## Conclusion

This research investigated the underlying mechanisms by which mindfulness is linked to BE/ME from the perspective of the mindfulness reperceiving theory. Why does mindfulness effectively inhibit ME and promote BE? A possible explanation is that mindful reperceiving enhances resilience, internal locus of control, and self-esteem. Future research can design mindfulness interventions and improve resilience from the view of internal locus of control and self-esteem to inhibit ME and promote BE.

## Data availability statement

The original contributions presented in the study are included in the article/supplementary material, further inquiries can be directed to the corresponding author/s.

## Ethics statement

The studies involving human participants were reviewed and approved by the paper was Ethics Committee of the Hunan Normal University. All procedures carried out in this study involving human participants were in accordance with the ethical standards of the Helsinki Declaration. All participants provided informed consents before completing the questionnaires, and were paid after completing the whole questionnaires. The patients/participants provided their written informed consent to participate in this study.

## Author contributions

XD: paper writing, paper revising, and data analysis. XL and XJ: paper revising. YX: study design and paper revising. All authors contributed to the article and approved the submitted version.

## Funding

This work was supported by the Philosophy and Social Science Project of Hunan Province of China (18YBA324).

## Conflict of interest

The authors declare that the research was conducted in the absence of any commercial or financial relationships that could be construed as a potential conflict of interest.

## Publisher's note

All claims expressed in this article are solely those of the authors and do not necessarily represent those of their affiliated organizations, or those of the publisher, the editors and the reviewers. Any product that may be evaluated in this article, or claim that may be made by its manufacturer, is not guaranteed or endorsed by the publisher.
